# Two-phase microalgae cultivation for RAS water remediation and high-value biomass production

**DOI:** 10.3389/fpls.2023.1186537

**Published:** 2023-06-12

**Authors:** Valeria Villanova, Jonathan Armand Charles Roques, Bita Forghani, Kashif Mohd Shaikh, Ingrid Undeland, Cornelia Spetea

**Affiliations:** ^1^ Department of Biological and Environmental Sciences, University of Gothenburg, Gothenburg, Sweden; ^2^ Department of Biological, Chemical and Pharmaceutical Sciences and Technologies (STEBICEF), University of Palermo, Palermo, Italy; ^3^ SWEMARC, The Swedish Mariculture Research Center, University of Gothenburg, Gothenburg, Sweden; ^4^ Department of Life Sciences-Food and Nutrition Science, Chalmers University of Technology, Gothenburg, Sweden

**Keywords:** Carotenoids, *Chlorella*, *Nannochloropsis*, *Phaedactylum tricornum*, proteins, PUFA, RAS wastewater, two-phase cultivation

## Abstract

The overall goal of this study was to provide solutions to innovative microalgae-based technology for wastewater remediation in a cold-water recirculating marine aquaculture system (RAS). This is based on the novel concept of integrated aquaculture systems in which fish nutrient-rich rearing water will be used for microalgae cultivation. The produced biomass can be used as fish feed, while the cleaned water can be reused, to create a highly eco-sustainable circular economy. Here, we tested three microalgae species *Nannochloropis granulata* (*Ng*), *Phaeodactylum tricornutum* (*Pt*), and *Chlorella sp* (*Csp*) for their ability to remove nitrogen and phosphate from the RAS wastewater and simultaneously produce high-value biomass, i.e., containing amino acids (AA), carotenoids, and polyunsaturated fatty acids (PUFAs). A high yield and value of biomass were achieved for all species in a two-phase cultivation strategy: *i)* a first phase using a medium optimized for best growth (f/2 14x, control); *ii)* a second “stress” phase using the RAS wastewater to enhance the production of high-value metabolites. *Ng* and *Pt* performed best in terms of biomass yield (i.e., 5-6 g of dry weight, DW.L^-1^) and efficient cleaning of the RAS wastewater from nitrite, nitrate, and phosphate (i.e., 100% removal). *Csp* produced about 3 g L^-1^ of DW and reduced efficiently only nitrate, and phosphate (i.e., about 76% and 100% removal, respectively). The biomass of all strains was rich in protein (30-40 % of DW) containing all the essential AA except Methionine. The biomass of all three species was also rich in PUFAs. Finally, all tested species are excellent sources of antioxidant carotenoids, including fucoxanthin (*Pt*), lutein (*Ng* and *Csp*) and *β*-carotene (*Csp*). All tested species in our novel two-phase cultivation strategy thus showed great potential to treat marine RAS wastewater and provide sustainable alternatives to animal and plant proteins with extra added values.

## 1 Introduction

Over the last 40 years, aquaculture has become one of the fastest-developing food-production activities worldwide (FAO, 2022). To satisfy the growing demands for fish with high nutritional values (i.e., high content of proteins and long chain omega-3 (n-3) polyunsaturated fatty acids, LC n-3 PUFAs), the aquaculture sector needs sustainable development. Two of the main current bottlenecks encountered by this industry are the treatment of the wastes produced by the fish, and the need for fish sustainably produced feed (Lenzi, 2013).

The intensification of the aquaculture industry mostly using open-water systems has led to some environmental concerns, such as the eutrophication caused by the leakage of nitrogen-rich nutrients into the environment, (Pahri et al., 2015). Land-based closed containment systems such as recirculating aquaculture systems (RAS) are better alternatives as they allow for a high degree of water reuse as well as ensure better control of the farming practices (Van Rijn, 2013; Ahmad et al., 2021; Ahmed and Turchini, 2021; Øvrebø et al., 2022).

At present, in RAS, nitrifying bacteria convert the ammonium ( ${\text{NH}}_{4}^{\;+}$ ) produced by the fish into nitrate ( ${\text{NO}}_{3}^{\;-}$ ), *via* nitrite ( ${\text{NO}}_{2}^{\;-}$ ), in the presence of oxygen (O_2_). As a result, ${\text{NO}}_{3}^{\;-}$ can slowly accumulate over time and reach concentrations that could affect the fish’s health and welfare (Camargo et al., 2005; Roques, 2013; Van Rijn, 2013; Roques et al., 2021). High ${\text{NO}}_{3}^{\;-}$ can later be managed through the biological conversion of ${\text{NO}}_{3}^{\;-}$ to nitrogen gas (N_2_) in anaerobic biofilters with denitrifying bacteria, anammox bacteria, or by regular water exchanges (Chen, 2002; Preena et al., 2021; Micolucci et al., 2023). The use of microalgae as a filter could be a promising alternative or complement to the current water remediation techniques, and in addition the biomass obtained could, later, be valorised into animal feed or feed supplements (Tejido-Nuñez et al., 2019; Tossavainen et al., 2019; Villar-Navarro et al., 2022).

In the last decades, the demand for fish meal and oils, mainly produced from the catch of small pelagic fish species, as aquaculture feedstock has increased tremendously (World Bank, 2013; FAO, 2020). However, the massive use of fish at the base of the marine food chain has led to increased prices and a shortage of natural fish stocks. Rapidly, plant-derived protein and oils have therefore been introduced as fish feed ingredients. However, their utilization is limited by the presence of a wide variety of anti-nutritional substances (Francis et al., 2001). In addition, the production of plant-derived protein and oils for fish feed requires arable lands and freshwater, which are both limited and could be instead directly used for human consumption (Hardy, 2010; Flachowsky et al., 2017; FAO, 2020). Therefore, alternative technologies such as microalgae cultivation has great potential as an eco-sustainable source of fish feed (Camacho-Rodríguez et al., 2018).

Microalgae are currently used in the aquaculture sector as live feed for different marine organisms, such as zooplankton, molluscs, crustaceans, and some species of fish (Koyande et al., 2019). The interest for use of microalgae in the food sector is because some species are as rich in proteins as food sources of animal (e.g., meat, fish, eggs, and milk) and vegetable origin (e.g., soy, Bleakley and Hayes, 2017). Microalgae are also a source of LC n-3 PUFAs (e.g., eicosapentaenoic acid, EPA, and docosahexaenoic acid, DHA), important for both fish and human health. However, the cultivation and harvesting of large volumes of microalgae, as well as the extraction of the molecules of interest, are energy-consuming and expensive processes. For this reason, despite their abundant presence in nature, to date, only a few marine species are marketed and used in the food industry as biomass (e.g. including species from the genus *Nannochloropsis*, *Phaeodactylum*, and *Chlorella*) or as extracts (e.g., β- carotene, fucoxanthin, EPA, DHA, proteins) (Sathasivam et al., 2019). Microalgae are also a sustainable alternative to heterotrophic bacteria and chemicals in wastewater treatment. Indeed, some microalgae species can convert both inorganic and organic pollutants from wastewater into high-value molecules (Samer, 2015).

Here, we investigated the ability of three industrially relevant microalgae species; *Nannochloropsis granulata* (*Ng*), *Phaeodactylum tricornutum* (*Pt*) and *Chlorella sp* (*Csp*) to grow in wastewater from a RAS producing high-value metabolites and at the same time cleaning the water. *Ng* and *Pt* are marine microalgae able to produce biomass enriched in EPA-rich lipids if grown under specific conditions (Abida et al., 2015; Villanova et al., 2017; Cheregi et al., 2021; Villanova et al., 2022). *Csp* can grow and significantly reduce both inorganic nitrogen and phosphorus in various types of wastewater (Asadi et al., 2019; Lima et al., 2020). For this reason, a strain of the genus previously isolated from a Sicilian coastline *Csp* was also included in this study (Arena et al., 2021). Moreover, the three selected species are rich in high-value carotenoids such as fucoxanthin and β-carotene, whose concentrations vary with the growth conditions (Arena et al., 2021; Villanova et al., 2021b; Villanova et al., 2022). A two-phase cultivation strategy was applied to obtain high-yield and high-quality biomass. At the end of the 19-22 days cultivation, the biochemical composition of the biomass and the nutrient removal efficiency were determined and compared among the strains.

## 2 Materials and methods

### 2.1 Microalgal species and preculture cultivation

The microalgae species used in this study were *Ng, Pt*, and *Csp*, obtained from the Gothenburg University Marine Algal Culture Collection (GUMACC https://www.gu.se/en/marina-vetenskaper/about-us/algal-bankgumacc, accessed on 1 March 2023). The cultures were not axenic, but 100 µg L^-1^ of ampicillin was added at the beginning of the cultivation to control the bacterial growth.

Precultures were maintained in 100 mL flasks at 16°C, with a light intensity of about 20 µmol photons m^−2^ s^−1^ and a 12/12 h light/dark cycle. The medium used was natural seawater collected from a depth of 30 m at the Tjärnö Research Station (University of Gothenburg, Strömstad, Sweden) supplemented with 14-fold concentrated nutrients (f/2 14x) to obtain a high concentration of biomass (Villanova et al., 2022). The seawater was filtered using two 0.4 µm GF/F glass fibre filters, the salinity was adjusted with deionized water to 26 practical salinity units (PSU), and it was sterilized by autoclaving at 121°C for 20 min. Finally, the nutrient stock solution from the standard f/2 marine cultivation medium (NaNO_3_, NaH_2_PO_4_, microelements, vitamins, Guillard and Ryther, 1962) was sterilized with cellulose filter paper (with a pore size of 0.22 µm) and 14 mL of each stock solution was added to 1 L of autoclaved seawater.

### 2.2 RAS wastewater

RAS wastewater (10 L, **Table 1**) was collected from the aquarium facilities of the University of Gothenburg (Gothenburg, Sweden), hosting a pilot scale research and development facility for the development of land-based seawater RAS at low temperatures (ca. 10°C). The fish species in the marine RAS were rainbow trout (*Oncorhynchus mykiss*) and Atlantic wolffish (*Anarhichas lupus*). In June 2020, the RAS wastewater was first filtered and then stored in 5 L plastic containers at 4°C until use. A subsample of filtered water was used for physiochemical characterization. The pH and salinity were measured using a Multimeter (pHenomenal MU 6100 H, VWR International, Radnor, PA, USA). The subsample was subsequently frozen (-80°C) and sent for determination of ${\text{NH}}_{4}^{\;+}$ -N, ${\text{NO}}_{2}^{\;-}$ -N, ${\text{NO}}_{3}^{\;-}$ -N, and ${\text{PO}}_{4}^{\;3-}$ -P to an accredited laboratory (Eurofins, Linköping, Sweden). **Table 1** shows the physiochemical characterization of both RAS wastewater and f/2 14x before algae cultivation. Nitrogen is one of the most important nutrients for microalgae growth (Abida et al., 2015) for this reason the same concentration of NaNO_3_ in f/2 14x was added to RAS wastewater in one-phase cultivation.

**Table 1:** Physicochemical characteristics of the RAS wastewater and f/2 14x prior algae cultivation.

Parameter	Salinity (PSU)	pH	NH_4_^+^ (mg-N L^−1^)	NO_2_^−^ (mg-N L^−1^)	NO_3_^−^ (mg-N L^−1^)	PO_4_^3−^ (mg-P L^−1^)
Method	Multimeter	Multimeter	ISO 15923-1:2013 Annex B	SS-EN ISO 13395:1997	SS-EN ISO 13395:1997	ISO 15923-1:2013 Annex F
RAS wastewater	34	5.45	0.87	0.0021	100	70
f/2 14x medium	26	8	0	0	1050	70

### 2.3 One-phase cultivation

*Ng* and *Pt* were grown in f/2 14x or RAS wastewater added with 14-fold concentrated NaNO_3_ (14N) using a Multicultivator MC 1000 OD (Photon System Instruments, Drásov, Check Republic) in flasks containing 80 mL of liquid culture at 22°C with a constant light intensity of 100 µmol photons m^−2^ s^−1^ and with air bubbling. The cultures were grown in triplicates until the stationary phase was reached (i.e., 18 days).

### 2.4 Two-phase cultivation

**Figure 1:**
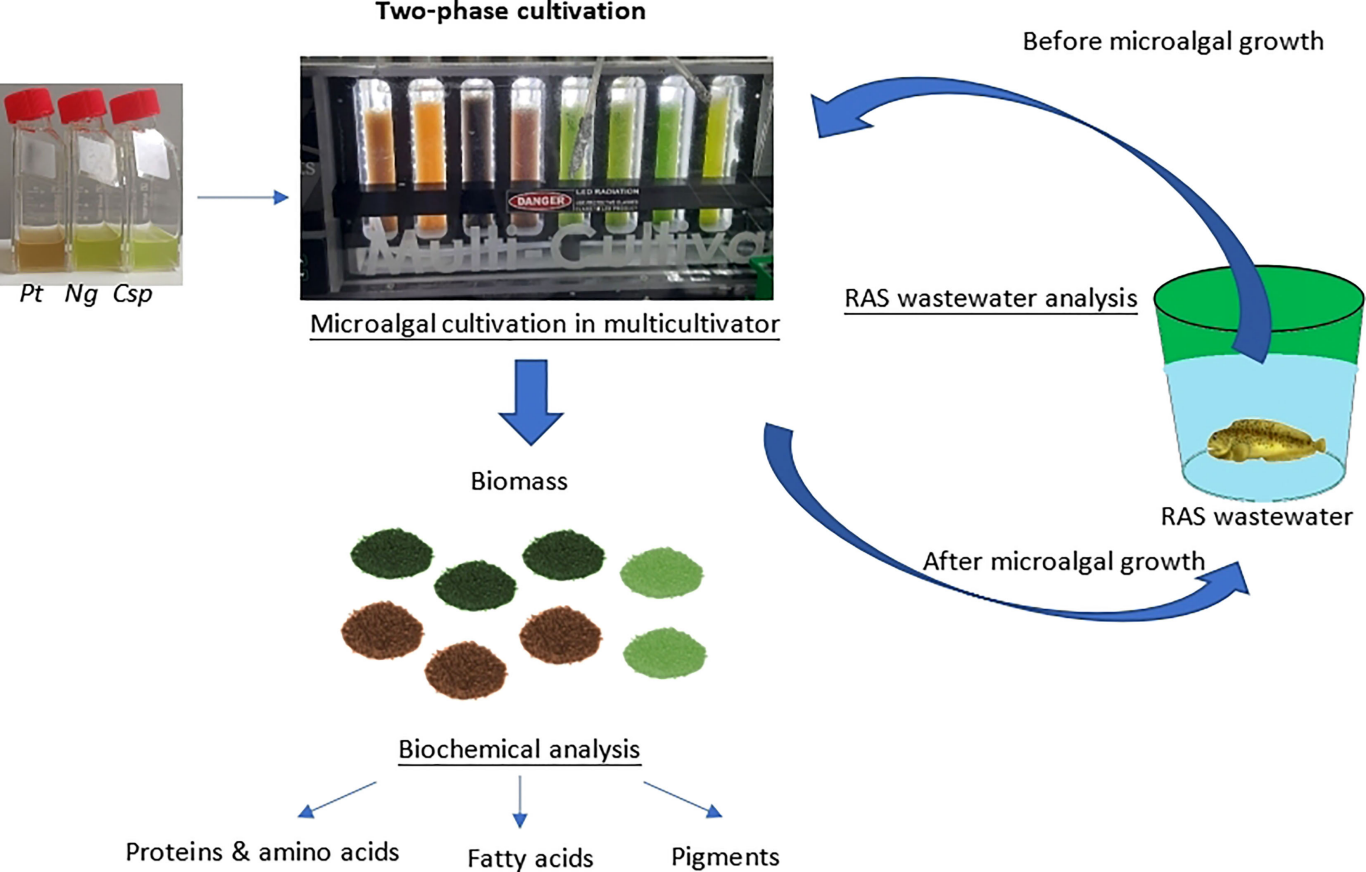
Overview of two-phase microalgae cultivation for cleaning of RAS wastewater and production of high-value biomass. *Ng*, *Nannochloropsis granulata*, *Pt*, *Phaeodactylum tricornutum*, and *Csp*, *Chlorella sp*.

*Ng*, *Pt*, and *Csp* were grown in the Multicultivator system described above. A two-phase-cultivation mode was used, which includes a first phase (phase I) using a medium and conditions for optimized growth to reach high biomass, and a second phase (phase II) using the RAS wastewater and stress conditions to stimulate the production of secondary metabolites. The experimental design of the two-phase cultivation is summarized in **Figure 1**. In phase I, the cultures were grown in f/2 14x at 22°C and the intensity of the light (cool white light) was increased over 19-22 days gradually from 100 to 800 µmol photons m^-2^ s^-1^, according to the specific algal growth performance. Phase I ended when the stationary phase was reached (19-22 days). In phase II, 40 mL cultures from phase I were inoculated in new flasks containing 40 mL of RAS wastewater. It contains inorganic nitrogen only in low concentrations and is relatively high in salinity, factors that can be stressful for microalgae growth but can produce high concentrations of certain molecules of interest, e.g., lipids and carotenoids. By contrast the phosphate concentration was the same in both 14x and RAS wastewater. These new cultures were grown at the same temperature as the RAS was maintained (10°C) and at a constant light intensity of 40 µmol photons m^−2^ s^−1^. The experiment ended when the stationary phase was reached (9-12 days). Four replicates of *Ng* and *Pt*, and two replicates of *Csp* were grown in parallel.

Algal growth was monitored every two days by measuring chlorophyll *a* fluorescence expressed in relative fluorescence units (RFU), using a Varioscan Flash Multimode Reader (Thermo Fisher Scientific, Vantaa, Finland), in a 96-well microplate. A total of 250 µL of each sample was added into separate wells of the microplate (in triplicate) and incubated for 10 min in darkness. Dilutions were performed when required (i.e., RFU > 30). Chlorophyll fluorescence was detected using a wavelength of 425 nm for excitation and 680 nm for emission (Cheregi et al., 2021; Villanova et al., 2022). In addition, the growth of both bacteria and algae was monitored as absorbance at 750 nm using a Thermo Scientific Evolution 60S UV-Visible Spectrophotometer (Thermo Fisher Scientific, Vantaa, Finland).

After the stationary phase was reached in both cultivation phases, the biomass yield was determined and expressed as g of dry weight (DW) L^-1^. A total of 5 mL of final cultures was filtered through pre-weighted dried GF/F (47 mm) Whatman filters (Cytiva, Marlborough, MA, USA), and then washed with 10 mL of 0.5 M ammonium carbonate to remove the excess salt. Finally, the filters containing the culture were incubated at 100°C for 24 h and weighed for DW determination according to the following formula:


$$DW\;\left(g\right)=\frac{\left(weight\;(filter+biomass,\;in\;g)\right)-\left(weight\;of\;filter,\;in\;g\right)}{0.005\;L\;\left(volume\;of\;filtered\;culture\right)}$$

Moreover, at the end of the two-phase cultivation, cells were collected by centrifugation and the pellets were immediately freeze-dried for further analysis.

### 2.5 RAS wastewater analysis before and after cultivation

On the first and last day of growth in phase II cultivation, cells were centrifuged for 5 min at 4000 *g* and the supernatant was collected and filtered. The supernatants were analysed immediately or preserved at -20°C until analysis.

The total nitrogen (TN) was calculated based on the concentrations of ${\text{NH}}_{4}^{\;+}$ , ${\text{NO}}_{2}^{\;-}$ , and ${\text{NO}}_{3}^{\;-}$ . Salinity and pH were measured using a multimeter (pHenomenal MU 6100 H, VWR International, PA, Radnor, USA). The ${\text{NH}}_{4}^{\;+}$ and ${\text{NO}}_{2}^{\;-}$ concentrations were determined using the powder pillow methods (salicylate method, 8155, and diazotization method, 8507, respectively, Hach-Lange, Dusseldorf, Germany) and the DR-2800 (Hach-Lange, Dusseldorf, Germany). The concentrations of ( ${\text{NO}}_{3}^{\;-}$ were determined using ion-exchange chromatography (HPLC 20A; Shimadzu, Kyoto, Japan) with a Shodex Asahipak NH2P-50 4D anion column (Showa Denko, Tokyo, Japan) and UV-VIS detector (SPD-20AV, Shimadzu) after filtration of samples through 0.2-μm pore-size PTFE membranes (Advantec, Tokyo, Japan) (Mojiri et al., 2020). The detection limits were 0.01, 0.002, and 0.5 mg-N L^−1^ for ${\text{NH}}_{4}^{\;+}$ , ${\text{NO}}_{2}^{\;-}$ and ${\text{NO}}_{3}^{\;-}$ , respectively. Finally, ${\text{PO}}_{4}^{\;3-}$ analysis was done using a commercial kit (114842 Spectroquant, Merck, Darmstadt, Germany) according to the manufacturer’s recommendations (detection limit: 0.5 mg-P P L^−1^). The data are presented as means ± standard deviation of four replicates of the supernatant from *Ng* and *Pt*, and two replicates of *Csp* and expressed as removal efficiency relative to the initial nutrient concentration (**Table 2**).

**Table 2:** Removal efficiency of total nitrogen (TN), *NH*_4_^+^, *NO*_2_^−^, *NO*_3_^−^ ad *PO*_4_^3−^ at the end of phase II.

Species	TN removal (%)	NH_4_^+^ removal (%)	NO_2_^−^ removal (%)	NO_3_^−^ removal (%)	PO_4_^3−^ removal (%)	
*Nannochloropsis granulata*	85.4 ± 11.4	41.2 ± 18.0	100.0 ± 0.0	100.0 ± 0.0	100.0 ± 0.0
*Phaeodactylum tricornutum*	86.7 ± 3.5	27.5 ± 14.8	100.0 ± 0.0	100.0 ± 0.0	100.0 ± 0.0
*Chlorella sp*	50.0 ± 2.8	-^*^	-^*^	75.7 ± 1.4	100.0 ± 0.0

Data are presented as means ± standard deviation of two to four biological replicates. ^*^Increase of in the nutrient concentration as compared to the initial condition.

### 2.6 Biochemical analysis of the biomass

#### 2.6.1 Protein content and amino acid composition

Freeze-dried biomass was bead-beaten for 2 min at 30 Hz (QIAGEN Tissuelyser II, Qiagen, Hilden, Germany) before the determination of total protein content. The total protein content of microalgal extracts was then determined by colorimetric analysis at 750 nm using the DC protein kit (Bio-Rad Laboratories, Hercules, CA, USA) following a sequential hot trichloroacetic acid (TCA) and alkaline extraction of the biomass (Slocombe et al., 2013). For the quantification, a standard curve of bovine serum albumin in the range of 0.225–1.35 mg L^-1^ was used.

For the determination of amino acid (AA) content, a known amount of freeze-dried biomass was resuspended in 4 mL of 6 N HCl in glass tubes followed by flushing with nitrogen gas for 30 s. The samples were then hydrolysed at 110°C for 24 h, after which they were filtered (syringe filter, PES, 0.2 μm) (VWR, Radnor, PA, USA) and diluted before AA determination using LC/APCI-MS as described previously (Forghani et al., 2022). All analyses were performed in duplicate.

#### 2.6.2 Fatty acid content and composition

Freeze-dried biomass was powdered and put into pre-weighed furnaced glass tubes. Fatty acids (FAs) were then extracted and methylated as previously described (Forghani et al., 2022). A known amount of powdered biomass was suspended in 400 μL of chloroform, and 200 μL of internal standard (i.e., heptadecanoic acid 100 μL mL^-1^) was added to the tubes. Samples were sonicated on ice for 1 h and transesterification was performed by adding 0.75 mL of HCl/MeOH (5% v/v) and incubating at 90°C for 90 min. After cooling, FA methyl esters (FAMEs) were extracted by adding 2 mL of hexane and mixing vigorously for 30 s followed by shaking at 300g for 20 min. The samples were then centrifugated at 2000g for 5 min and the upper phase was transferred into a clean tube. The extraction was repeated one more time for increasing the recovery of FAMEs. After the evaporation of hexane, measurement of FAMEs was carried out by using an Agilent Technologies 7890 A GC system connected to Agilent Technologies 5975 inert MSD (Kista, Sweden). Acquisition, identification, and quantification of FAME peaks were performed by their comparison with the 37-component FAME standard mix (Supelco, Bellefonte, PA, USA, Cavonius et al., 2014) by using Masshunter Quantitative Analysis software (version B.09.00, Agilent Technologies, Santa Clara, CA, USA). FA analyses were done in duplicate.

#### 2.6.3 Pigment composition

A known amount of freeze-dried biomass was mixed with 5 mL of 90% (v/v) acetone in falcon tubes covered with aluminium foil to prevent light penetration. The samples were ground in a glass homogenizer and incubated at 4°C for 4 h. After this period, the samples were centrifuged at 3000 g for 5 min. The supernatant was filtered using a filter with a pore size of 0.2 µm and used for pigment analysis. The pigment composition was obtained by using HPLC coupled with a PDA detector (Villanova et al., 2022). 100 µL of samples were analysed in a Shimadzu UFLC system (Shimadzu corporation, Kyoto, Japan) equipped with an Alltima C18 (RP18, ODS, Octadecyl) 150 × 4.6 mm column. The pigments were eluted through a low-pressure gradient system constituted by solvent A with methanol and 0.5 M ammonium acetate buffer (85:15, v/v), solvent B with acetonitrile and milliQ water (90:10, v/v), and solvent C with 100% ethyl acetate. The following program was used: 100% B:0% C: (8 min), 90% B:10% C: (8.6 min), 65% B:35% C (13.1 min), 31% B:69% C (21 min), and 100% B:0% C (27 min). The identification of pigments was done by comparison of their retention time and spectra with standards (DHI, Hørsholm, Denmark) run under the same conditions. The quantification of the pigment concentration was then obtained by comparing the area of the corresponding standard. The pigment concentration was then normalized for freeze-dried biomass and expressed as µg mg^-1^ of DW. Four replicates were processed for *Ng* and *Pt*, and duplicates for *Csp* were used.

#### 2.6.4 Statistical analysis

The biochemical composition of the biomass was compared among the different species using a two-way analysis of variance (ANOVA) test (GraphPad 9.5.1 Software, San Diego, CA, USA). *p*-values were used to quantify the variability among the three different species. Differences were considered significant for *p*-values< 0.05.

## 3 Results

### 3.1 Microalgal growth in f/2 and RAS wastewater

The physicochemical characteristics of the RAS wastewater and f/2 14x before the start of the algae cultivation were different (**Table 1**). For this reason, the RAS wastewater composition was slightly adjusted for optimal algae cultivation and the modified substrate named RAS wastewater 14N. As nitrogen compounds, the RAS wastewater contained ${\text{NH}}_{4}^{\;+}$ , ${\text{NO}}_{2}^{\;-}$ , and ${\text{NO}}_{3}^{\;-}$ , but the latter was much less abundant than in f/2 14x. Moreover, RAS wastewater was characterized by lower pH and slightly higher salinity than f/2 14x (**Table 1**). Therefore, the RAS water was supplemented with ${\text{NO}}_{3}^{\;-}$ and pH was adjusted to reach the same levels as in f/2 14x. In a one-stage cultivation, *Pt* and *Ng* were able to grow in undiluted RAS wastewater 14N similarly to f/2 14x, which can be explained by their similar nutrient composition (**Table 1**) after a lag phase during the first eight cultivation days (**Supplementary File 1A, B**). The biomass yields were similar, but *Pt* showed a higher yield than *Ng* in the tested conditions (i.e., 3 and 2 g DW L^-1^, respectively) (**Supplementary File 1C**).

**Figure 2:**
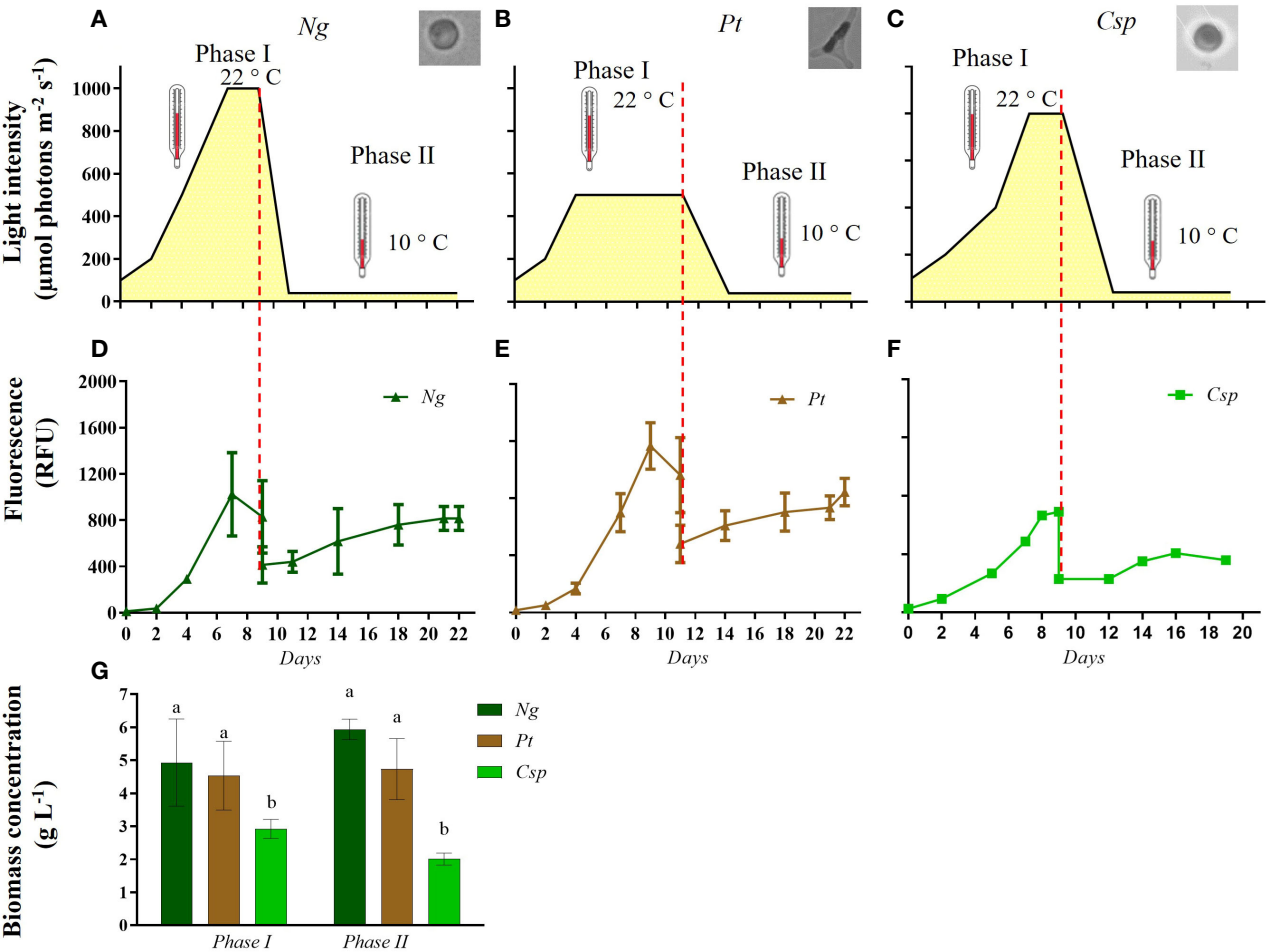
Growth conditions **(A–C)** and growth curve of **(D)** *Nannochloropsis granulata* (*Ng*, dark green line), **(E)** *Phaeodactylum tricornutum* (*Pt*, brown line), and **(F)** *Chlorella sp* (*Csp*, light green line) in a two-phase system in Multicultivator. **(G)** Biomass concentration obtained at the end of phase I and phase II cultivation in *Ng* (dark green bar), *Pt* (brown bar), and *Csp* (light green bar). Data shown in **(D–G)** are the means ± standard deviation of four biological replicates for *Ng* and *Pt*, and two biological replicates for *Csp*. Different letters indicate significant differences among the species (*p<* 0.05).

To reduce the lag phase and produce a high yield and value of biomass, the three microalgal strains were grown using a two-phase cultivation strategy. The growth conditions used for the different species in two-phase cultivation are shown in **Figure 2A-C**. **Figure 2D-F** shows the growth monitored as chlorophyll fluorescence (RFU) along the cultivation time in the two-phase cultivation. *Ng* and *Pt* grew better than *Csp* in phase I. Moreover, after the 1:1 dilution in RAS wastewater in phase II, *Ng* and *Pt* reached a similar RFU as at the end of phase I. In contrast, *Csp* in phase II was not able to recover the maximum RFU obtained in phase I. The dry weight (DW) of the biomass was determined at the end of phase I and phase II (**Figure 2G**). *Ng* and *Pt* reached significantly higher DW than *Csp* (5-6 and 3 g L^-1^, respectively) in phase I. Moreover, both *Pt* and *Ng* yielded similar DW at the end of phase II. In contrast, *Csp* produced only about 2 g DW L^-1^ after phase II, confirming previous observations on growth profiles. This can be explained by the fact that *Chlorella* species are mostly freshwater microalgae, hence not adapted to the high salinity of f/2 and RAS wastewater (i.e., 26 and 34 PSU, respectively) (Darienko et al., 2019).

### 3.2 Biochemical composition of the biomass

**Figure 3:**
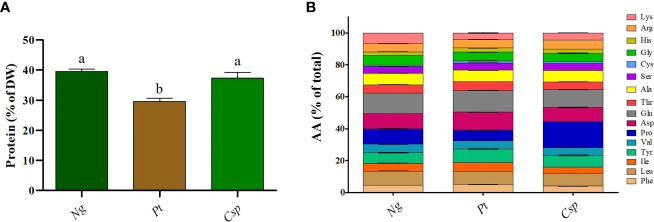
Protein content **(A)** and amino acid profile **(B)** of *Nannochloropsis granulata* (*Ng*, dark green bar), *Phaeodactylum tricornutum* (*Pt*, brown bar), and *Chlorella sp* (*Csp*, light green bar) grown in two-phase system in Multicultivator. Data shown are the means ± standard deviation of four biological replicates for *Ng* and *Pt*, and two biological replicates for *Csp*. Different letters indicate significant differences among the species (*p<* 0.05).

To determine the industrial potential of the tested microalgae species as fish feed, a biochemical analysis of the biomass collected at the end of phase II was performed. In particular, to test the microalgae as an eco-sustainable alternative to terrestrial animal and plant proteins, the protein content and AA profile were determined. The biomass of *Ng* and *Csp* contained about 40% protein of DW as compared to 30% in *Pt* (**Figure 3A**). The proteins from the all three species contained all essential AA (i.e., Arginine, Arg; Histidine, Hys; Isoleucine, Ile; Leucine, Leu; Lysine, Lys; Phenylalanine, Phe; Threonine, Thr; and Valine, Val) except for Methionine (Met) and Tryptophane (Trp), the latter, which is not captured by the applied method. Only slight differences in the content of essential AA were detected between the three species. Both *Ng* and *Pt* contained Glutamic acid (Glu) and Aspartic acid (Asp) as main AA, with about 13-14% and 10-11% of the total, respectively. *Csp* contained Proline (Pro) and Glu as the main amino acids, with about 16% and 11 % of the total, respectively (**Figure 3B**).

**Figure 4:**
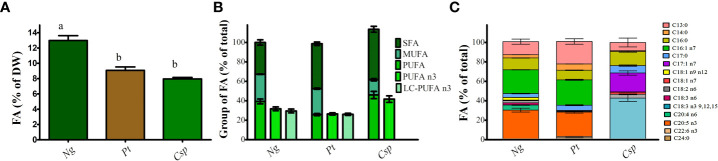
Fatty acid content **(A)**, Saturated, monounsaturated and polyunsaturated fatty acid **(B)** and fatty acid profile **(C)** of *Nannochloropsis granulata* (*Ng*, dark green bar), *Phaeodactylum tricornutum* (*Pt*, brown bar), and *Chlorella sp* (*Csp*, light green bar) grown in a two-phase cultivation in Multicultivator. Data showed are the means ± standard deviation of biological replicates for *Ng* and *Pt*, and two biological replicates for *Csp*. Different letters indicate significant differences among the species (*p*< 0.05).

FA content and profile were also analyzed due to their importance in both fish and human nutrition. The highest FA content was obtained in *Ng* followed by *Pt* and *Csp* with about 13, 9, and 8 % of DW, respectively (**Figure 4A**). FAs can be classified as saturated (SFAs), monounsaturated (MUFAs), and PUFAs to indicate the presence of only carbon single bonds, one double bond, and two or more double bonds respectively. SFAs were more abundant in *Pt* and *Csp* (i.e., about 50% of total FA) as compared to *Ng* (i.e., about 30% of total FA). *Pt* and *Ng* showed higher MUFAs (i.e., about 27-28% of the total) than *Csp* (i.e., about 16% of the total). Finally, *Ng* showed the highest content of PUFAs followed by *Csp* and *Pt* with 39, 32, and 27% of the total, respectively (**Figure 4B**). The FA profile was similar for *Ng* and *Pt* and dominated by C13:0 (i.e., about 13 and 23% of TFA, respectively), C16:0 (i.e., about 12 and 10 %, respectively), C16:1 (i.e., about 24 and 26%, respectively), and EPA (i.e., 25-30%). The main FAs in *Csp* were C16:0 (i.e., about 10% of TFA), C17:1 (i.e., about 13%), and alpha-linolenic acid (C18:3 n-3, i.e., about 42%). Moreover, *Pt* also contained a low concentration of DHA (about 1%) (**Figure 4C**). The relative content of n-3 PUFAs was about 30, 26, and 29%, respectively in *Ng*, *Pt*, and *Csp*. The corresponding percentages of LC n-3 PUFAs (i.e., EPA+DHA) were about 29 and 26%, in *Ng* and *Pt*, respectively. LC n-3 PUFAs were not detected in *Csp*.

**Figure 5:**
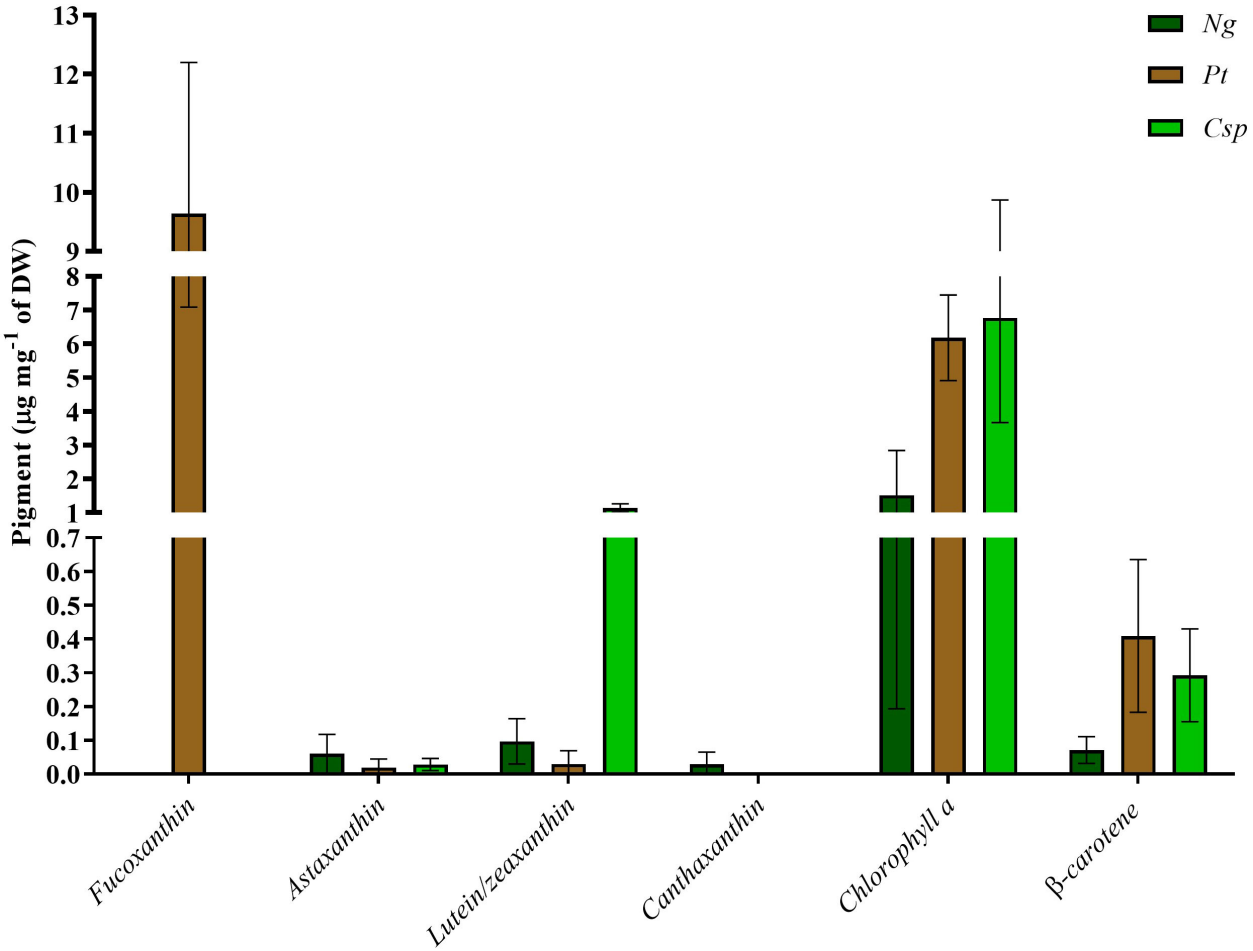
Pigment profile of *Nannochloropsis granulata* (*Ng*, dark green bar), *Phaeodactylum tricornutum* (*Pt*, brown bar), and *Chlorella sp* (*Csp*, light green bar) grown in a two-phase system in Multicultivator. Data shown are the means ± standard deviation of four biological replicates for *Ng* and *Pt*, and two biological replicates for *Csp*. Different letters indicate significant differences among the species (*p<*0.05).

Finally, the pigment content was analyzed as an important source of antioxidants for stabilization of the microalgae biomass or products derived thereof. Also, some studies have revealed importance of antioxidants in animal and human nutrition (Miyashita et al., 2011; Tan and Hou, 2014; Petrushkina et al., 2017; Dawood et al., 2018). *Pt* had as main pigments chlorophyll *a*, fucoxanthin, and *β*-carotene with about 6, 10, and 0.4 μg per mg of DW, respectively. *Ng* was characterized by about 1.5 μg of chlorophyll *a*, 0.01 μg of lutein/zeaxanthin, and 0.07 μg of *β*-carotene per mg of DW. *Ng* also showed traces of astaxanthin and canthaxanthin. Finally, *Csp* contained chlorophyll *a*, lutein/zeaxanthin, and *β*-carotene as main pigments with about 6, 1, and 0.3 μg per mg of DW, respectively (**Figure 5**). *Csp* also produced a small amount of astaxanthin. Raw data for all biochemical analyses are available in **Supplementary File 2**.

### 3.3 Nutrient removal from RAS wastewater

To determine the potential of the different microalgae species to clean the RAS wastewater, the removal efficiency of each nitrogen compound was calculated at the end of phase II. *Ng* and *Pt* performed best in terms of cleaning the RAS wastewater from ${\text{NO}}_{2}^{\;-}$ , ${\text{NO}}_{3}^{\;-}$ (i.e., 100% removal) as compared to *Csp* which reduced about 76% of ${\text{NO}}_{3}^{\;-}$ and none of the ${\text{NO}}_{2}^{\;-}$ . ${\text{NH}}_{4}^{\;+}$ was reduced by 41.2 and 27.5% in *Ng* and *Pt*, respectively. The concentration of ${\text{NH}}_{4}^{\;+}$ was instead increased in *Csp* at the end of phase II, indicating some different nitrogen degradation pathways in *Chlorella* species. The sum of nitrogen compounds ( ${\text{NH}}_{4}^{\;+}$ , ${\text{NO}}_{2}^{\;-}$ , ${\text{NO}}_{3}^{\;-}$ ) was not equal to the total nitrogen (TN) in all the tested samples, indicating that many organic nitrogen compounds (e.g., protein and AA origin) are released in the medium by the microalgae. Finally, all tested species efficiently and completely removed all ${\text{PO}}_{4}^{\;3-}$ contained in the RAS wastewater (**Table 2**).

## 4 Discussion

In this study, we demonstrate the ability of three microalgal species (i.e., *Ng*, *Pt*, and *Csp*) to grow in RAS wastewater. Our results are in line with previous studies for *Nannochloropsis, Phaeodactylum tricornutum*, and *Chlorella* species, along with other species (Sirakov and Velichkova, 2014; Tejido-Nuñez et al., 2019; Villar-Navarro et al., 2021). However, *Pt*, *Ng*, and *Csp* showed about 10, 22, and 2.5-fold, respectively, higher biomass productivity than related species grown in RAS wastewater to date (**Table 3**). These results demonstrate the importance of two-phase cultivation to increase algal production capabilities in terms of high-quality and quantity of biomass. This novel strategy is based on the concept that each microalgae species grows best in certain (optimal) conditions, which however do not necessarily correspond to the conditions for the highest production of molecules of interest (e.g., PUFAs and carotenoids). Here, we grew *Ng*, *Pt*, and *Csp* in the following conditions: *i)* Phase I: each species was cultivated at 22°C with a gradual increase along growth to avoid photoinhibition phenomena (Aléman-Nava et al., 2017; Ali et al., 2021; Karpagam et al., 2022) and in enriched medium (i.e., f/2 14x); *ii)* phase II: the cells were transferred to a medium containing RAS wastewater in stress conditions (e.g., low nutrients, low temperature, high salinity) to increase the production of molecules of interest. Similar strategies were previously used for *Nannochloropsis oculata*, *Pt*, and *Chlorella vulgaris* resulting in increased lipid and carotenoid productivity (Sirakov and Velichkova, 2014; Villar-Navarro et al., 2021). To our knowledge, this is the first time that a two-phase cultivation strategy was applied for growing microalgae in RAS wastewater. Moreover, we show that by using this strategy the algal biomass yield can increase in *Ng* and *Pt* by a factor of about 3 and 2, respectively, as compared to one-phase cultivation (**Supplementary File 1C**; **Figure 2G**).

**Table 3:** Comparison of microalgal biomass productivity from this study with previous studies in RAS wastewater.

Species	Biomass productivity (mg DW L^−1^ d^−1^)	Cultivation condition	Reference
*Nannochloropsis granulata*	270 ± 13	Two-phase cultivation	This study
*Phaeodactylum tricornutum*	225 ± 49	Two-phase cultivation	This study
*Chlorella sp*	105 ± 7	Two-phase cultivation	This study
*Nannochloropsis gaditana*	23 ± 1	Batch cultivation	(Villar-Navarro et al., 2021)
*Phaeodactylum tricornutum*	32 ± 1	Batch cultivation	(Villar-Navarro et al., 2021)
*Chlorella vulgaris*	42.6	Continuous cultivation	(Gao et al., 2016)

Microalgae are good candidates to partially replace fishmeal and fish oil in fish feed. For instance, inclusions levels of 7.5-30% of *Nanochloropsis oceanica* extracts gave promising results in cold-water species such as Atlantic salmon (*Salmo salar*) and spotted wolffish (*Anarhichas minor*) (Sørensen et al., 2017; Gong et al., 2018; Knutsen et al., 2019). However, only a few studies focused on the biomass composition of microalgae grown in RAS wastewater to date (Sirakov and Velichkova, 2014; Villar-Navarro et al., 2021). Here, we determined the content of proteins, AA, FA, and carotenoids in the biomass derived from phase II cultivation of *Ng*, *Pt*, and *Csp* to evaluate their potential as alternative sustainable fish feed. We found a higher protein content in all tested microalgae (30-40% of DW) than previous results obtained in other species or strains grown in similar conditions (i.e., 14-37% of DW) Cho and Kim, 2011). However, all these values are significantly lower than the average protein content found in fish feed (i.e., 60-72%, Cho and Kim, 2011), calling for downstream up-concentration of the proteins. This can be done for example with the pH-shift process commonly applied to e.g. soybeans and peas (Dumoulin et al., 2021), but also to algae (Cavonius et al., 2015; Trigo et al., 2023). It is well known that under nitrogen deplete conditions (i.e., conditions found in phase II of this work), protein concentration can be reduced in several microalgae species, explaining our results (Jia et al., 2015; Canelli et al., 2020; Latsos et al., 2020). Moreover, the proteins of *Pt*, *Ng* and *Csp* were constituted by almost all essential AA, confirming previous results for closely related species (Villar-Navarro et al., 2021; Forghani et al., 2022).

*Ng, Pt*, and *Csp* showed higher PUFAs content as compared to related species when grown in RAS wastewater (Villar-Navarro et al., 2021; Forghani et al., 2022). The increase in PUFAs can be explained by the use of low temperatures (i.e., 10°C) during phase II cultivation, as concentration of PUFAs generally decreases at increasing temperatures in microalgae, including in *Pt* (Qiao et al., 2016; Santin et al., 2021). The biomasses were also rich in n-3 PUFAs, i.e., 25-30% of FAs were constituted by EPA in *Pt* and *Ng* and by C18:3 n-3 in *Csp.* These amounts were higher than the reference values for fish oil, confirming the potential of these microalgal strains as a substitute for fish feed (Villar-Navarro et al., 2021).

Finally, the pigment content of the microalgae biomass was evaluated based on their beneficial effect on animals and humans for blocking macrophage-mediated inflammation and inflammation-induced obesity in both *in-vivo* and *in-vitro* assays (Tan and Hou, 2014; Petrushkina et al., 2017). Fucoxanthin is the most abundant pigment carotenoid in diatoms and can make up to 1-2.5 % of DW (Yi et al., 2015; Guo et al., 2016). A similar fucoxanthin content was found in *Pt* in our study and was 3-fold higher than in previous results for the same species (Villanova et al., 2021b). This finding can be explained by the use of low light intensity in phase II, often correlated to an increase in the fucoxanthin content in *Pt* (Khaw et al., 2022). Other valuable carotenoids detected in *Pt*, *Csp*, and *Ng* were β-carotene and lutein/zeaxanthin, confirming previous results (Serra et al., 2021; Villanova et al., 2022). It is known that β-carotene concentration content can increase in microalgae cultivated under salt stress (Villanova et al., 2021a). The salinity in the tested RAS wastewater was only slightly higher than in the microalgae cultivation medium (**Table 1**). This can explain why we did not detect a significant increase in the concentration content of this carotenoid.

The last part of this work was focused on the determination of the capability of *Ng*, *Pt*, and *Csp* to remove the nutrients present in RAS wastewater. In RAS, ${\text{NH}}_{4}^{\;+}$ is oxidized into ${\text{NO}}_{3}^{\;-}$ *via* ${\text{NO}}_{2}^{\;-}$ by nitrifying bacteria in a biofilm reactor. All these compounds can accumulate over time in RAS and if not appropriately managed through regular water changes or denitrification, may negatively affect the fish. High ${\text{NH}}_{4}^{\;+}$ is neurotoxic for fish (Wilkie, 2002). ${\text{NO}}_{2}^{\;-}$ converts hemoglobin into methemoglobin, which is not capable to bind O_2_ (Russo and Thurston, 1977; Williams and Eddy, 1986). ${\text{NO}}_{3}^{\;-}$ toxicity is thought to be similar to that of ${\text{NO}}_{2}^{\;-}$ , but to a lower extent (Stormer et al., 1996; Camargo et al., 2005). As most of the RAS nowadays are partial RAS (i.e., without denitrification), ( ${\text{NO}}_{3}^{\;-}$ can slowly accumulate over time and reach concentrations which could affect fish health and welfare (Russo and Thurston, 1977; Wilkie, 2002). High ${\text{NO}}_{3}^{\;-}$ can be managed through the biological conversion of ${\text{NO}}_{3}^{\;-}$ to nitrogen gas (N_2_) in anaerobic denitrifying biofilters, or by regular water exchanges (Williams and Eddy, 1986; Camargo et al., 2005). The concentration of ${\text{NH}}_{4}^{\;+}$ , ${\text{NO}}_{2}^{\;-}$ and ${\text{NO}}_{3}^{\;-}$ measured in our RAS wastewater are quite typical for a conventional RAS with only nitrification, with ${\text{NH}}_{4}^{\;+}$ values below 1 mg-N L^−1^, ${\text{NO}}_{2}^{\;-}$ below 0.5 mg-N L^−1^ and accumulation of ${\text{NO}}_{3}^{\;-}$ up to 100-1000 mg-N L^−1^ (Brazil et al., 1996; Krumins et al., 2001; Van Rijn and Ebeling, 2007; Roques, 2013; Ciji and Akhtar, 2020; Sikora et al., 2022). Our results showed that all three microalgae species were able to remove efficiently ${\text{NO}}_{3}^{\;-}$ from their environment. In particular, *Pt* and *Ng* were able to completely remove ${\text{NO}}_{3}^{\;-}$ and ${\text{NO}}_{2}^{\;-}$ and are therefore the two most promising candidates to treat RAS wastewater. Despite a decent removal efficiency of ${\text{NO}}_{3}^{\;-}$ (75.7%), *Csp* did not remove any other nitrogenous waste compounds, and even increased the concentration of ${\text{NH}}_{4}^{\;+}$ and ${\text{NO}}_{2}^{\;-}$ . This relatively low performance could again be linked to the fact that *Csp* is a mostly freshwater microalgae species and its performance in this study was probably affected by the exposure to the relatively high salinity of both f/2 and RAS wastewater (Darienko et al., 2019). The ${\text{NH}}_{4}^{\;+}$ removal efficiencies of *Pt* and *Ng* were also quite limited (27.5 and 41.2%, respectively), which was quite expected as ${\text{NO}}_{3}^{\;-}$ seems to be the preferred substrate of these microalgae. ${\text{NH}}_{4}^{\;+}$ seems to inhibit the uptake of ${\text{NO}}_{3}^{\;-}$ in *Pt* (Cresswell and Syrett, 1979). Yongmanitchai and Ward (1991) used ${\text{NH}}_{4}^{\;+}$ , ${\text{NO}}_{3}^{\;-}$ and urea as nitrogen sources for *Pt*, showing that the growth was inhibited in the culture supplemented with ${\text{NH}}_{4}^{\;+}$ alone or in combination with ${\text{NO}}_{3}^{\;-}$ or urea *Nannochloropis* species can use either ${\text{NH}}_{4}^{\;+}$ , ${\text{NO}}_{2}^{\;-}$ or ${\text{NO}}_{3}^{\;-}$ as the sole nitrogen source, but ${\text{NO}}_{3}^{\;-}$ and ${\text{NO}}_{2}^{\;-}$ seem to be the preferred substrates for these species, as introduced ${\text{NH}}_{4}^{\;+}$ acidify the pH conditions of the medium (Sauer et al., 2001; Liu et al., 2017). As a result, *Pt* and *Ng* are great candidates to remove ${\text{NO}}_{3}^{\;-}$ from marine RAS wastewater, but they should be used in combination with other treatment solutions to also remove excess ${\text{NH}}_{4}^{\;+}$ (e.g., nitrifying bacteria).

To conclude, all species tested in this study showed great potential as a sustainable alternative to fish oil and meal and as a source of antioxidants for fish feed. These species also showed great potential as a multifunctional vegan protein ingredient for various food products in which also n-3 PUFAs and antioxidants are wanted. Moreover, the two-phase cultivation can be used as a strategy to *i)* increase the productivity and content of high-value molecules in the biomass of the tested strains, and *ii*) recycle the RAS wastewater. Different growth conditions and microbial species should be tested to further optimize this process.

## Data availability statement

The original contributions presented in the study are included in the article/**Supplementary Material**. Further inquiries can be directed to the corresponding authors.

## Author contributions

VV, JR, and CS contributed to the conception and design of the study. VV performed the experiments. VV, JR, BF, and KS performed sample analyses. VV performed the statistical analyses. IU and CS supervised the project. VV, JR, IU, and CS secured funding. VV and JR wrote the first draft of the manuscript. All authors contributed to the manuscript revision, and read, and approved the submitted version.

## Supplementary material

Supplementary File 1 Growth curve and final biomass of microalgal strains from GUMACC collection in one-phase cultivation. Growth curve of **(A)** *Phaeodactylum tricornutum* (*Pt*, brown lines) and **(B)** *Nannochloropsis granulata* (*Ng*, dark green lines) in f/2 14x (continuous lines) and RAS wastewater 14N (dotted lines). **(C)** Final biomass concentration of *Ng* and *Pt* grown in both f/2 14x and RAS wastewater 14N. Data shown are the means ± standard deviation of three biological replicates.

Supplementary File 2 Biochemical analysis of the biomass produced in phase II cultivation.
