# Supplementary figures and images for: Two-phase microalgae cultivation for RAS water remediation and high-value biomass production
Source: Front Plant Sci. 2023 Jun 12;14:1186537. doi: 10.3389/fpls.2023.1186537 (PMC10292630; doi:10.3389/fpls.2023.1186537)

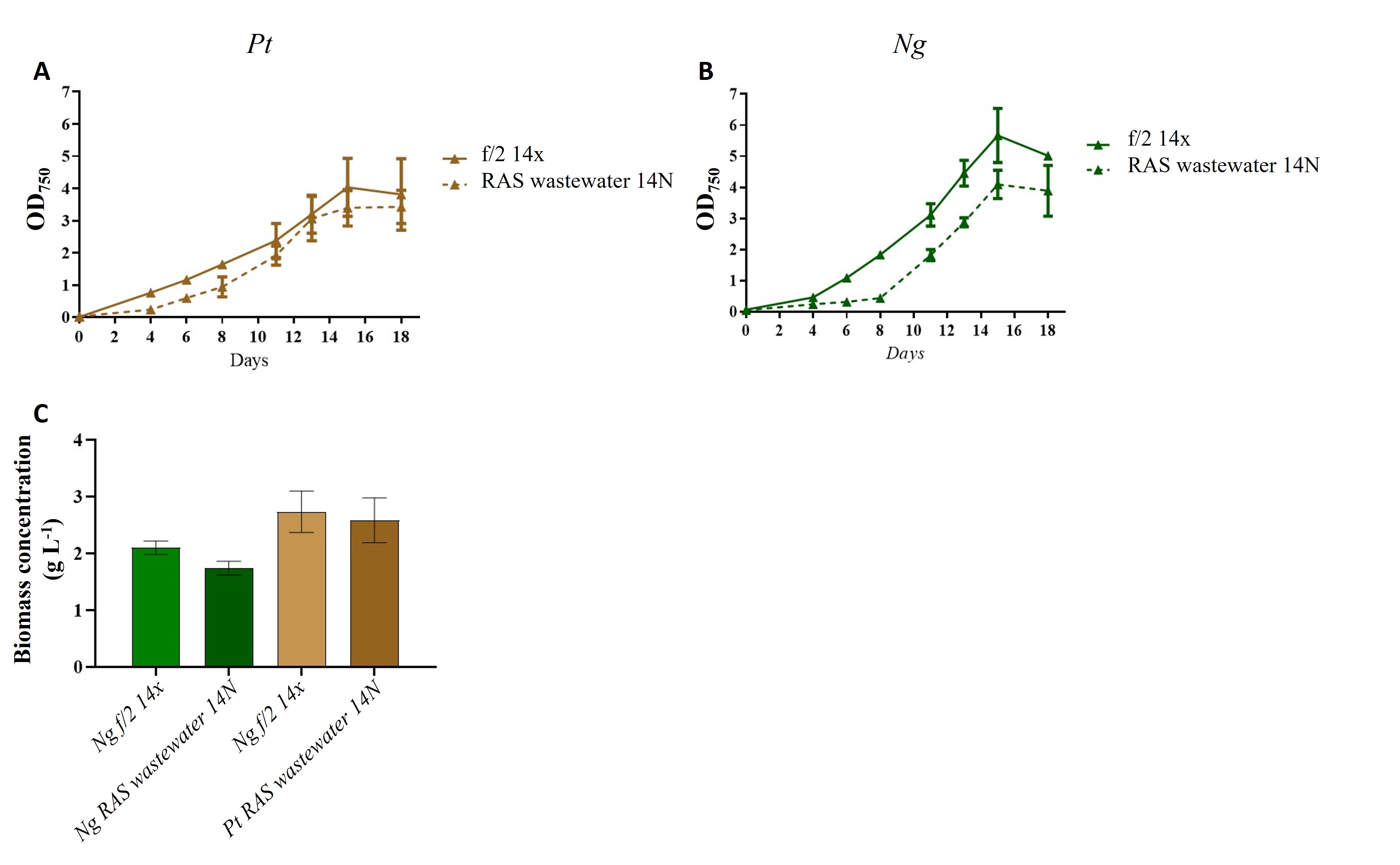

Supplement: Supplementary File 1 — Growth curve and final biomass of microalgal strains from GUMACC collection in one-phase cultivation. Growth curve of (A) Phaeodactylum tricornutum (Pt, brown lines) and (B) Nannochloropsis granulata (Ng, dark green lines) in f/2 14x (continuous lines) and RAS wastewater 14N (dotted lines). (C) Final biomass concentration of Ng and Pt grown in both f/2 14x and RAS wastewater 14N. Data shown are the means ± standard deviation of three biological replicates. [file DataSheet_1.zip › Supplementary File 1.jpg]
